# Surgical resection for liver metastasis and local recurrence of pheochromocytoma 16 years after primary surgery: A case report

**DOI:** 10.1016/j.ijscr.2021.105712

**Published:** 2021-02-27

**Authors:** Mineto Ohta, Atsushi Fujio, Shigehito Miyagi, Kazuaki Tokodai, Wataru Nakanisihi, Michiaki Unno, Takashi Kamei

**Affiliations:** Department of Surgery, Graduate School of Medicine, Tohoku University, Japan

**Keywords:** PCC, pheochromocytoma, MIBG, metaiodobenzylguanidine, SDHC, succinate dehydrogenase complex subunit B, PASS, pheochromocytoma of the adrenal gland scaled score, Phaeochromocytoma, Liver metastasis, Surgical resection, Case report

## Abstract

**Introduction and importance:**

Pheochromocytomas arise from the adrenal medulla and are rare. Pheochromocytomas metastasize to bone, lung and liver, and surgery might be the curative treatment. However, few cases are detected when they are resectable lesions because of difficulty in diagnosis and rapid growth. We herein report a patient who underwent resection of liver metastasis and local recurrence of pheochromocytoma.

**Case presentation:**

A 74-year-old woman visited our hospital for treatment for liver and retroperitoneal tumors. She had undergone left adrenal gland resection for pheochromocytoma 16 years earlier. Eleven years after primary surgery, breast cancer was diagnosed and resected. During the breast cancer follow-up, a liver tumor was identified with computed tomography. Breast cancer recurrence and metastasis were considered, so chemotherapy was administered first. However, the liver tumor gradually enlarged, and another lesion appeared in the retroperitoneum. The tumors were diagnosed as pheochromocytoma recurrence using ^123^I-metaiodobenzylguanidine scintigraphy, and she underwent resection of the local recurrence and liver metastasis. She was discharged on postoperative day 25 without complications, and no evidence of recurrence occurred more than 3.5 years postoperatively.

**Clinical discussion:**

All pheochromocytomas have metastatic potential; however, there are no reliable markers to predict malignancy. Early detection of recurrence by regular imaging and complete resection are important in the treatment. If the recurrence was oligometastasis and tumor growth is slow, surgical resection may be eligible.

**Conclusion:**

A favorable outcome resulted from complete resection for liver metastasis and local recurrence of pheochromocytoma.

## Highlights

• Pheochromocytoma is a rare tumor with malignant potential.
• Distant metastases are almost always unresectable, and carry a poor prognosis.
• Early detection and complete resection resulted in good outcomes.

## 1 Introduction

Pheochromocytoma (PCC) is a rare tumor originating in the adrenal medulla, and the prevalence in patients with hypertension is 0.1%–0.6% [1]. PCC usually causes symptoms of catecholamine excess and is often discovered incidentally during imaging examinations. All PCCs have malignant potential, and the metastatic rate is 10%–30% [2]. The most common sites of metastasis are the lymph nodes, lung, bone, and liver [3], and when a recurrent lesion is found, it is often in an advanced and unresectable state. The 5-year survival of patients with unresectable metastases is less than 50% [4]. Currently, surgical resection might be the curative treatment for patients with locally recurrent or distant resectable metastases. In patients with unresectable PCC, the main therapeutic goals are tumor reduction and control of catecholamine excess [5]. Here, we report a patient who underwent resection for liver metastasis and local recurrence of pheochromocytoma 16 years after the primary surgery.

## 2 Case presentation

**Fig. 1:**
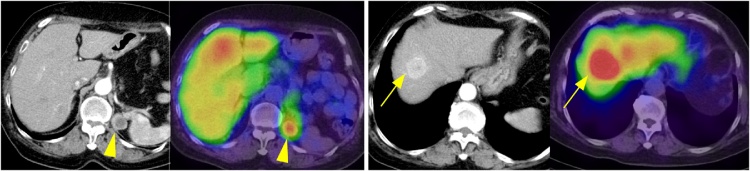
Contrast-enhanced CT and ^123^I MIBG scintigraphy findings. CT image showing ring-enhanced tumors in the left retroperitoneal area (arrowhead) and liver (arrow). Uptake of metaiodobenzylguanidine (^123^I-MIBG) in these tumors was confirmed during scintigraphy (arrow). CT, computed tomography.

**Fig. 2:**
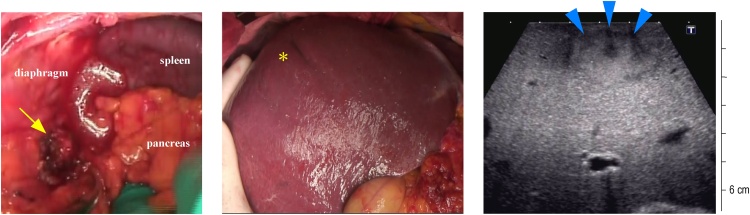
Operative findings for the locally recurrent and liver tumors. The retroperitoneal recurrent tumor (arrow) was adhered to the diaphragm and greater psoas muscle. The liver tumor was located in hepatic segment VIII, and the tumor was accompanied by dimpling (asterisk) and was confirmed as a hyperechoic tumor (arrowhead) by ultrasonography.

**Fig. 3:**
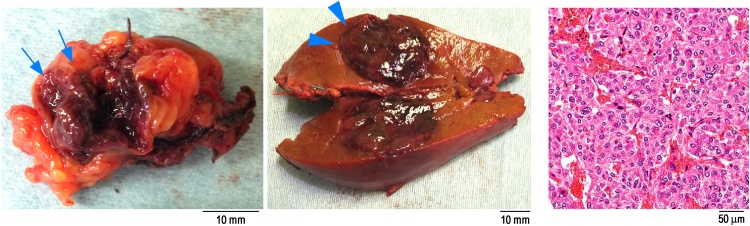
Findings in the resected specimen and pathology. The images are ospecimens of the retroperitoneal (arrow) and liver (arrowhead) tumors. Both tumors were brownish and consistent with recurrence of pheochromocytoma. Hematoxlin eosin staining showed zellballen growth pattern. There were trabecular and alveolar growing tissue with round-shaped nucleus and basophilic cytoplasm.

A 74-year-old woman visited our hospital to under resection of liver and retroperitoneal tumors. She had a surgical history of left adrenalectomy for PCC and total right mastectomy for breast cancer, 16 years and 5 years earlier, respectively. The liver tumor was identified on computed tomography (CT) during the breast cancer follow-up and was initially considered liver metastasis from breast cancer. Although chemotherapy for breast cancer was administered, the liver tumor slowly progressed, and another mass appeared in the retroperitoneum near the left crus of the diaphragm (Fig. 1). The patient’s urine noradrenaline concentration was slightly elevated (278 μg/mL) and ^123^I-metaiodobenzylguanidine (MIBG) accumulated in the liver and retroperitoneal tumors in MIBG scintigraphy (Fig. 1). She was diagnosed with local recurrence and liver metastasis of PCC and underwent surgery. Resection of the locally recurrent tumor was performed first. The pancreatic tail and spleen were mobilized, and the tumor was resected with the greater psoas muscle and diaphragm. For the liver metastatic lesion, the tumor was accompanied by dimpling, and the tumor was confirmed as hyperechoic by intraoperative ultrasonography (Fig. 2). Partial resection (segment VIII) was performed. At the time of tumor removal, blood pressure decreased due to endogenous catecholamine deficiency, but the next day, blood pressure became normal. Both tumors were brownish in appearance and pathologically diagnosed as PCC recurrence (Fig. 3). The patient’s postoperative course was good, and the number of antihypertensive drugs was reduced. She was discharged 25 days postoperatively without complications, and there was no evidence of recurrence more than 3.5 years after the surgery. This report is compliant with the SCARE 2020 criteria published on case report submissions [6].

## 3 Discussion

The conditions ‘benign pheochromocytoma’ and’ malignant pheochromocytoma’ were combined to ‘pheochromocytoma’ in the 2017 WHO classification of endocrine tumors; all pheochromocytomas have metastatic potential [7]. The most common sites of distant metastasis are the lymph nodes (36%), lung (47%), bone (64%), and liver (32%) [3], and the 5-year survival of patients with unresectable metastases is less than 50% [4]. Patients with visceral metastases have a poorer prognosis than those with isolated bone disease [7]. There is no standard protocol for the treatment of metastatic PCC. Currently, surgical resection might be the curative treatment for patients with locally recurrent or distant resectable metastasis. However, most patients are not amenable to complete surgical removal. This is because there are no established predictors for recurrence and many patients do not receive adequate follow-up care after resection of the primary tumor [2,8]. Someindicators; a large tumor size, elevated plasma or urine methoxytyramine, pathological pheochromocytoma of adrenal gland scaled score (PASS) > 6, loss of tumoral succinate dehydrogenase complex subunit B (SDHB) staining in immunohistochemistry, and Ki-67 proliferation index > 3% [[9], [10], [11]] were used as evaluation for malignancy, however, these indicators do not necessarily correlate with prognosis. For the patients with unresectable recurrence, the main therapeutic goals are tumor reduction and controlling the symptoms caused by catecholamine excess. There are several palliative options: radiation therapy, radiofrequency, cryoablation, transarterial chemoembolization, and systemic chemotherapy [5,12].

In our case, metastatic lesions appeared in the liver more than 10 years after primary surgery for PCC. The histopathological findings after the first surgery identified no necrosis, mitosis, or vascular invasion (PASS score < 6). Moreover, blood pressure and plasma noradrenaline levels normalized, postoperatively. The risk of recurrence was considered low, and imaging follow-up was completed 5-years after surgery. The subsequent liver metastasis was found incidentally at an early stage during the breast cancer follow-up. It is considered that the completely resection was possible because of the oligometastasis and slow growth of recurrent tumors. From the histopathology of the recurrent tumor, and because the Ki67 labelling index was 4% and invasion to the psoas major muscle was present, we considered this a highly malignant tumor. Preoperatively, the patient was taking four antihypertensive drugs: doxazosin mesilate, atenolol, olmesartan medoxomil, and amlodipine besilate, and postoperatively, her symptoms were controllable with only nifedipine and amlodipine besilate. Her postoperative course is currently 3.5 years in length, without recurrence. Since there is a possibility of recurrence, careful follow-up including images is conducted. Early diagnosis and complete resection may be effective to achieve a good long-term prognosis in our and similar patients.

## 4 Conclusion

Liver metastasis and local recurrence of PCC was detected at an early stage. If the recurrence was oligometastasis and tumor growth is slow, surgical resection may be eligible.

## Declaration of Competing Interest

The authors report no declarations of interest.

## Sources of funding

The authors declare that they have no supportive funding.

## Ethical approval

This work does not require a deliberation by the ethics committee.

## Consent

Written informed consent was obtained from the patients for publication of this case report and any accompanying images. A copy of the written consent is available for review by the Editor-in-Chief of this journal on request.

## Author contribution

Mineto Ohta is a major contributor in writing the manuscript. Atsushi Fujio, Shigehito Miyaagi, Kazuaki Tokodai, Michiaki Unno, and Takashi Kamei were attending doctors who performed clinical treatment, including surgical operation. All authors have read and approved the final manuscript.

## Registration of research studies

Not applicable.

## Guarantor

Mineto Ohta accept full responsibility for the work and had controlled the decision to publish.

## Provenance and peer review

Not commissioned, externally peer-reviewed.
